# Amylopectin Copolymers Grafted with RAFT-Obtained Synthetic Polymers: Synthesis and Aqueous Solution Behavior

**DOI:** 10.1021/acs.biomac.5c02198

**Published:** 2026-02-10

**Authors:** Melinda-Maria Bazarghideanu, Marius-Mihai Zaharia, Ana-Maria Macsim, Marcela Mihai, Stergios Pispas

**Affiliations:** 54574 Petru Poni Institute of Macromolecular Chemistry, 41A Grigore Ghica Voda Alley, Iasi 700487, Romania

## Abstract

According to the requirements of modern society for durable biomaterials with multiple functionalities, the principal target of this study was to apply the “grafting to” scheme on the synthesis of several hybrid copolymers based on amylopectin (AMP) and poly­(*N*-isopropylacrylamide) (PNIPAM), poly­(oligo­(ethylene glycol) methyl ether methacrylate) (POEGMA), and poly­(2-(dimethylamino) ethyl methacrylate) (PDMAEMA) as synthetic polymer components, thus obtaining hybrid copolymers responsive to different stimuli, such as temperature, pH, and ionic strength. The synthetic polymers were synthesized by reversible addition–fragmentation chain transfer polymerization and contain reactive carboxyl groups attached to one end of the polymeric chains. The successful synthesis of novel graft copolymers AMP-*g*-PNIPAM, AMP-*g*-PDMAEMA, and AMP-*g*-POEGMA was confirmed by ATR-FTIR and ^1^H NMR spectroscopies, by evidencing the appearance of new aliphatic ether bonds as a consequence of covalent grafting of the synthetic polymers onto the AMP chains. The pH, temperature, and ionic strength responsiveness of the newly obtained copolymers’ aqueous solutions were followed by dynamic and electrophoretic light scattering analysis, revealing the intra/interchain self-assembly depending on the ionizable groups present in their structure, according to their protonation or deprotonation equilibria.

## Graphical abstract

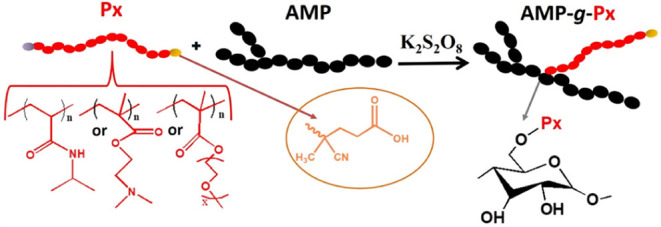

## Introduction

In recent years, natural polymers such as polysaccharides received considerable attention in a wide range of application areas, mainly due to their already renowned features and properties, such as nontoxicity, biodegradability, and biocompatibility.^1,2^ Besides their multifaceted properties, polysaccharides also present some disadvantages, such as low mechanical resistance, insolubility in certain solvents, and sensitivity to high temperatures.^3,4^ Therefore, following the current society’s needs for resistant and stable biomaterials with adapted functionalities, the most reliable solution is to combine polysaccharides with synthetic polymers, aiming to create hybrid macromolecular materials that integrate the functionalities and the properties of the used building blocks. Also, the increasing interest in developing new biomaterials based on polysaccharides and synthetic polymers relies on the desire to reduce the production and use of petroleum-based polymeric materials and to exploit natural resources.^5,6^ Amylopectin (AMP), which constitutes roughly 70–80% of starch, is a highly branched polysaccharide with an average molecular weight between 10^4^ and 5·10^4^ g·mol^–1^. It consists of many α-d-glucopyranose units linked together in a dense branching arrangement by α-(1 → 4) and α-(1 → 6) glycosidic bonds. Due to its favorable biodegradability, biocompatibility, low cost, and abundance in nature, AMP is increasingly being considered as a potential substrate for a wide variety of bioapplications. However, the practical use of native AMP, with its attractive biodegradability and biocompatibility, is limited due to the inadequate mechanical strength, lack of stimuli-response capabilities, and low solubility in water,^7–9^ which require the modification of AMP through either physical or chemical means to enhance its function and processability. The numerous free hydroxyl groups located along the AMP natural polymer chain provide many reactive sites for various chemical transformations on AMP. Consequently, AMP can be chemically modified with ester,^10^ ether,^11,12^ and cross-linked^13^ derivatives, as well as by grafting^14,15^ synthetic polymers onto AMP. Grafting synthetic polymers onto AMP provides a flexible and versatile approach for modifying the properties of polysaccharides while retaining the beneficial properties of the native polysaccharide. Grafting strategies are divided into the following categories: “grafting through”, “grafting from”, and “grafting to”.^16^ The “grafting through” method occurs during copolymerization of the macromonomer with the forming of a branched structure due to the end group that is able to polymerize, while the “grafting from” method involves the generation of active initiating sites randomly along the polysaccharide backbone that are used to grow chains of polymer. The “grafting to” strategy refers to attaching presynthesized polymers that have functional end groups to polysaccharides, resulting in a high degree of control over the properties of the obtained copolymers.^16^ Reversible addition–fragmentation chain-transfer (RAFT) polymerization provides polymer chemists with a unique set of advantages when designing AMP-based graft copolymers. RAFT allows for the production of polymers with controlled molecular weight distribution and narrow dispersity and provides control over the functionality of the polymer life cycle,^16,17^ all of which are critical to optimizing grafting efficiency and controlling the macromolecular architecture produced upon grafting. The ability to produce and utilize a well-defined functional synthetic polymer by RAFT polymerization provides the capacity to fine-tune the structure and properties of the hybrid copolymer produced upon grafting (e.g., length of the RAFT-synthesized polymer, density of grafting, and type of chemical composition), and ultimately how the resulting copolymer will behave in an aqueous solution.^18–21^ The grafting of RAFT-derived polymer chains that are hydrophilic, thermoresponsive, or pH-responsive significantly enhances the water solubility, colloidal stability, and functional versatility of AMP. At the same time, these polymer chains impart to the AMP new and unique stimuli-responsive properties that are not available in the native polysaccharide. Using the “grafting to” approach with RAFT-prepared polymers also allows to obtain additional controls over the synthetic grafted component and the polysaccharide backbone independently, making it easier to evaluate systematic structure–property correlations.^22,23^ Therefore, grafting RAFT-derived polymers onto AMP is an excellent way to convert a naturally abundant branched polysaccharide into versatile advanced biomaterials that can be used in aqueous and biologically relevant environments.

Recently, a variety of stimuli-responsive polymers that respond to one or a number of stimuli in triggered ways, thermally, pH, redox, ionic strength, and any combination of these, have been developed using some of the available polymerization methods on various macromolecular architectures. These polymers include linear, grafted, block, star-shaped, and network polymers produced using controlled/living radical polymerization methods, as well as a combination of ring-opening and step-growth synthesis.^24–26^ All of these developments have provided advanced materials for multiple applications, including biomedical polymers, smart coatings, and polymers for separation and environmental remediation technologies.^27–29^ A particular area of increased interest is the combination of synthetic stimuli-responsive polymers with natural polymers, such as polysaccharides. This combination brings together the responsiveness and chemical modifications of synthetic polymers and the biocompatibility, biodegradability, and biofunctionality of polysaccharide backbones. Within the methods of controlled radical polymerization, RAFT polymerization has been used extensively to produce stimuli-responsive polymers^30–32^ with high levels of precision to control molecular mass, composition, and functionality, as well as to graft those polymers to many different types of polysaccharides, mainly via the “grafting from” technique, producing hybrid materials that are suitable for wastewater treatment, drug delivery, and many other applications. Numerous studies have proven that both the ATRP and RAFT-based “grafting from” techniques can be successfully employed to attach an array of synthetic polymers to a wide variety of amino and hydroxyl groups containing polysaccharides (e.g., AMP).^33–35^ However, while the functionalization of synthetic polymers using RAFT has many advantages, e.g., tolerance for a wide variety of functional groups and ease of modification at the ends of the polymer chains, the use of RAFT-synthesized polymers in “grafting to” techniques to modify polysaccharides is still relatively under-represented.

The present study examines AMP modifications made through a “grafting to” method, where three temperature- or pH-sensitive water-soluble polymers (synthesized using RAFT-polymerization) were utilized: (1) poly­(*N*-isopropylacrylamide) (PNIPAM), (2) poly­(oligo­(ethylene glycol) methyl ether methacrylate) (POEGMA), and (3) poly­(2-(dimethylamino)­ethyl methacrylate) (PDMAEMA). All three homopolymers were chosen due to the existing knowledge of their temperature- and/or pH-sensitive behavior as well as because their solubility behavior is tunable. This increased variety of structural and behavioral motifs allows for better-designed and more efficient AMP-based hybrid copolymers than was described in a previous study only with PDMAEMA, which is the only one of these three polymers commonly used in conjunction with the ATRP-based “grafting from” method (as indicated in literature^36^). PNIPAM possesses LCST near physiological conditions (31–33 °C) depending on whether the hydrogen bonding of amino-ester or hydrophobic interactions of nonionic isopropyl groups dominates the local environment. PNIPAM is commonly used as an in vitro model polymer for studies of protein-like thermoresponsiveness and biomedical applications. The superior biocompatibility of PDMAEMA has facilitated the grafting of the polymer onto many polysaccharides, including dextran,^25^ chitosan,^32,37^ alginate,^38^ starch,^39^ chondroitin sulfate,^40^ and cellulose,^41^ with the goal of producing thermoresponsive hydrogels for use in medicine.^42^ PDMAEMA is a cationic polymer that has dual responsiveness to temperature and pH, with a typical p*K*_a_ value between 7 and 7.5. At pH values less than p*K*_a_, the tertiary amine groups are protonated, making the polymer highly hydrophilic. At pH values greater than p*K*_a_, the tertiary amine groups are deprotonated, and therefore, the polymer is more hydrophobic.^43–45^ At physiological pH (i.e., pH 7.4), PDMAEMA is partially protonated and thus able to form both electrostatic and hydrogen bonding interactions with many types of biomacromolecules, such as nucleic acids, proteins, peptides, and drugs, which makes PDMAEMA particularly useful for biomedical applications and drug delivery.^46,47^ The POEGMA family is a versatile group of graft-like polymers consisting of polymethacrylate backbone with oligo­(ethylene glycol) side chains.^48,49^ POEGMA’s water solubility and thermoresponsiveness are very sensitive to the length and density of the oligo­(ethylene glycol) side chains.^45,50,51^ Generally, the POEGMA polymer has greater water solubility when the oligo­(ethylene glycol) units are longer. On the other hand, when the oligo­(ethylene glycol) units are comparatively shorter, they can exhibit LCST-type thermoresponsive behavior. The POEGMA-based polymers have received a great deal of attention in the medical community, mainly because of their outstanding biocompatibility, low toxicity, and antifouling properties, and thus, POEGMA-based polymers have emerged as one of the largest classes of studied materials that demonstrate stimulated responsiveness for biomedical applications.^52–54^

Designing polysaccharide-based hybrid materials that meet both environmental and programmable demands is one of the main challenges faced by biomacromolecules. Although amylopectin contains natural properties such as biodegradability and biocompatibility, its lack of inherent properties that are triggered by stimuli limits its applications in advanced aqueous and biological systems. The use of “grafted polymer systems” to modify AMPs has been studied in the academic community quite extensively; however, these methods have been widely regarded as providing limited control of the architecture and chemical uniformity of the grafts added to the AMP backbone. A more precise manner to engineer AMP-based hybrid macromolecules could be achieved by means of a “grafting to” strategy, by the incorporation of defined stimuli-responsive synthetic polymer chains on AMP backbones. Reversible addition–fragmentation chain-transfer (RAFT) polymerization methods that provide controlled molecular weight and narrow dispersity to polymers have made it possible to design graft copolymers based on the predictable solution behavior of those polymers. Consequently, the purpose of this study is to present a versatile platform for attaching thermoresponsive and pH-responsive polymer chains to the AMP backbone via RAFT methods. When combined with the natural properties of AMPs, a unique opportunity will exist to establish direct relationships between the structure of AMP and its ability to maintain structure, self-assemble, and respond to the environment in an aqueous solution. The successful synthesis of AMP-grafted copolymers onto RAFT polymers was confirmed through the use of ATR-FTIR spectroscopy, ^1^H NMR spectroscopy, and TGA analysis. In addition, the behavior of these copolymers in aqueous solution as a function of pH, temperature, and ionic strength was also systematically studied by using DLS and ELS techniques. The end products of this work were designed to incorporate the beneficial properties of the branched polysaccharide material (AMP) while introducing new tunable environmental responses (i.e., selected environmental responses such as temperature, pH, etc.) to create hybrid polymeric composite (HP) hybrid systems operating as smart biomacromolecular systems. According to our knowledge, to date, this is the first report of using a “grafting to” method to produce hybrid graft copolymers of amylopectin with stimuli-responsive RAFT-synthesized polymers. This approach serves as a framework for designing responsive polysaccharide-polymeric hybrid materials, creating new possibilities for the development of functional coatings, nanoscale materials, and bioderived delivery systems with enhanced performance characteristics.

## Materials and Methods

### Materials

Maize AMP, 2,2′-azobis­(isobutyronitrile) (AIBN, 99%), potassium persulfate (99%), 1,4-dioxane, *n*-hexane, 4-cyano-4-[(dodecylsulfanylthiocarbonyl)­sulfanyl]­pentanoic acid (CDSPA, ≥96.5%), 4-cyano-4-(phenylcarbonothioylthio)­pentanoic acid (CPPA, 99.1%), *N*-isopropylacrylamide (NIPAM, powder, ≥99%), and the dialysis tubing cellulose membrane (molecular cutoff 12000–14000 Da) were purchased from Sigma-Aldrich (Sigma Chemical Co.; St. Louis, MO, USA) and used as received. The oligo­(ethylene glycol) methyl ether methacrylate) (OEGMA, 99.6%, *M*_n_ = 500 g mol^–1^) and 2-(dimethylamino)­ethyl methacrylate stabilized with hydroquinone monomethyl ether (DMAEMA, 99.6%) were also obtained from Sigma-Aldrich and were passed through an inhibitor-removing column (inhibitor remover for removing hydroquinone and monomethyl ether hydroquinone). Other materials used (without further purification) were dimethyl sulfoxide (DMSO, ≥99.7%, from Lach-Ner, Ltd., Továrn, Neratovice, Czech Republic), hydrochloric acid (HCl, ≥36.5%, from Chemical Company SA, Iasi, Romania), and ethanol (EtOH, 95%, from Chemical Company SA, Iasi, Romania). The aqueous solutions were prepared in ultrapure water (conductivity of 0.552 μS/cm) (EVOQUA Ultra Clear TPTWF Systems, Evoqua Water Technologies LLC; Barsbüttel, Germany).

### RAFT Synthesis of the Synthetic Polymers

The PNIPAM polymer (Đ = 1.11, *M*_n_ = 2900 g·mol^–1^, Figure S1) was synthesized via RAFT polymerization, using a previously published protocol.^32^ PDMAEMA (Đ = 1.1, *M*_n_ = 1148 g·mol^–1^, Figure S1) and POEGMA (Đ = 1.13, *M*_n_ = 7414 g·mol^–1^, Figure S1) were synthesized similarly, in accordance with the procedures previously published.^55^ Briefly, the synthetic homopolymers PNIPAM, PDMAEMA, and POEGMA were obtained using the corresponding monomers in dioxane as the solvent, with AIBN being used as the radical polymerization initiator. For RAFT polymerization, two chain transfer agents were used: CDSPA for the NIPAM and OEGMA polymerization and CPPA for DMAEMA polymerization. Following the RAFT polymerization procedure, the polymers were precipitated in *n*-hexane and dried under vacuum, and their structure was spectroscopically established by ATR-FTIR (Figure S2) and ^1^H NMR (Figure S3).

#### PNIPAM

ATR-FTIR (cm^–1^): 2870–2970 (υ_C–H_), 1638 (υ_C=O_), 1531 (υ_N–H_), 1454–1360 (υ_C–H_), 1171 (υ_C–N_); ^1^H NMR (δ, ppm, DMSO): 1.09 (−CH­(C**H**_3_)_2_), 3.9 (−C**H**(CH_3_)_2_), 1.3–1.59 (−CH–C**H**_2_−), 2.02 (−C**H**–CH_2_−).

#### PDMAEMA

ATR-FTIR (cm^–1^): 2773–2824 (υ_C–H_), 1723 (υ_C=O_), 1237 (υ_C–O_), 1147 (υ_O=C—O—C_), 1455–1390 (υ_C–H_), 1101 (υ_C–N_); ^1^H NMR (δ, ppm, DMSO): 0.8–1.2 (−CH_2_–C–C**H**_3_), 1.5–1.7 (−C**H**_2_–C–CH_3_), 2.2–N­(C**H**_3_)_2_, 4.1 (−C**H**_2_C**H**_2_N).

#### POEGMA

ATR-FTIR (cm^–1^): 2800–3000 (υ_C–H_), 1726 (υ_C=O_), 1096 (υ_C–O–C_), 1450 and 1348 (υ_C–H_), 1246 (υ_—O=C—O—_); ^1^H NMR (δ, ppm, DMSO): 0.8–1.2 (−CH_2_–C–C**H**_3_), 1.8 (−C**H**_2_–C–CH_3_), 3.25 (−(C**H**_2_CH_2_O)_*x*_–C**H**_3_), 3.3–3.7 (−(C**H**_2_C**H**_2_O)_*x*_–CH_3_), 4.1 (O–C**H**_2_CH_2_–O).

### Synthesis of AMP-Graft Copolymers

The synthesis of AMP-*g*-PNIPAM, AMP-*g*-PDMAEMA, and AMP-*g*-POEGMA copolymers was performed following the “grafting to” technique and according to a previously reported protocol,^32^ by linking the synthetic polymers to polysaccharides via a covalent coupling reaction using potassium persulfate as the source of chain end reactivating radicals (Scheme 1). Amylopectin (0.5 g, corresponding to 3.086·10^–3^ moles of anhydroglucose units) was first solubilized in a mixture of ethanol, NaOH 10 wt % aqueous solution, and H_2_O (1:2:10 vol %) (30 mL) and then mixed with the aqueous solution of the RAFT-obtained synthetic polymer (0.3 g/5 mL): PNIPAM (0.103·10^–3^ moles), PDMAEMA (0.261·10^–3^ moles), or POEGMA (0.404·10^–4^ moles), (0.3 g PNIPAM, PDMAEMA, or POEGMA) and the initiator (potassium persulfate, 6.4 mg/1 mL, 2.36 × 10^–5^ moles). The molar ratios between anhydroglucose units of branched amylopectin and synthetic RAFT polymer chains were approximately 30:1 for PNIPAM, 12:1 for PDMAEMA, and 76:1 for POEGMA. The reaction was carried out in a nitrogen inert gas atmosphere, at 50 °C, and under stirring for 24 h. The obtained copolymers were purified by dialysis at room temperature in ultrapure water and finally freeze-dried.

**Scheme 1:**
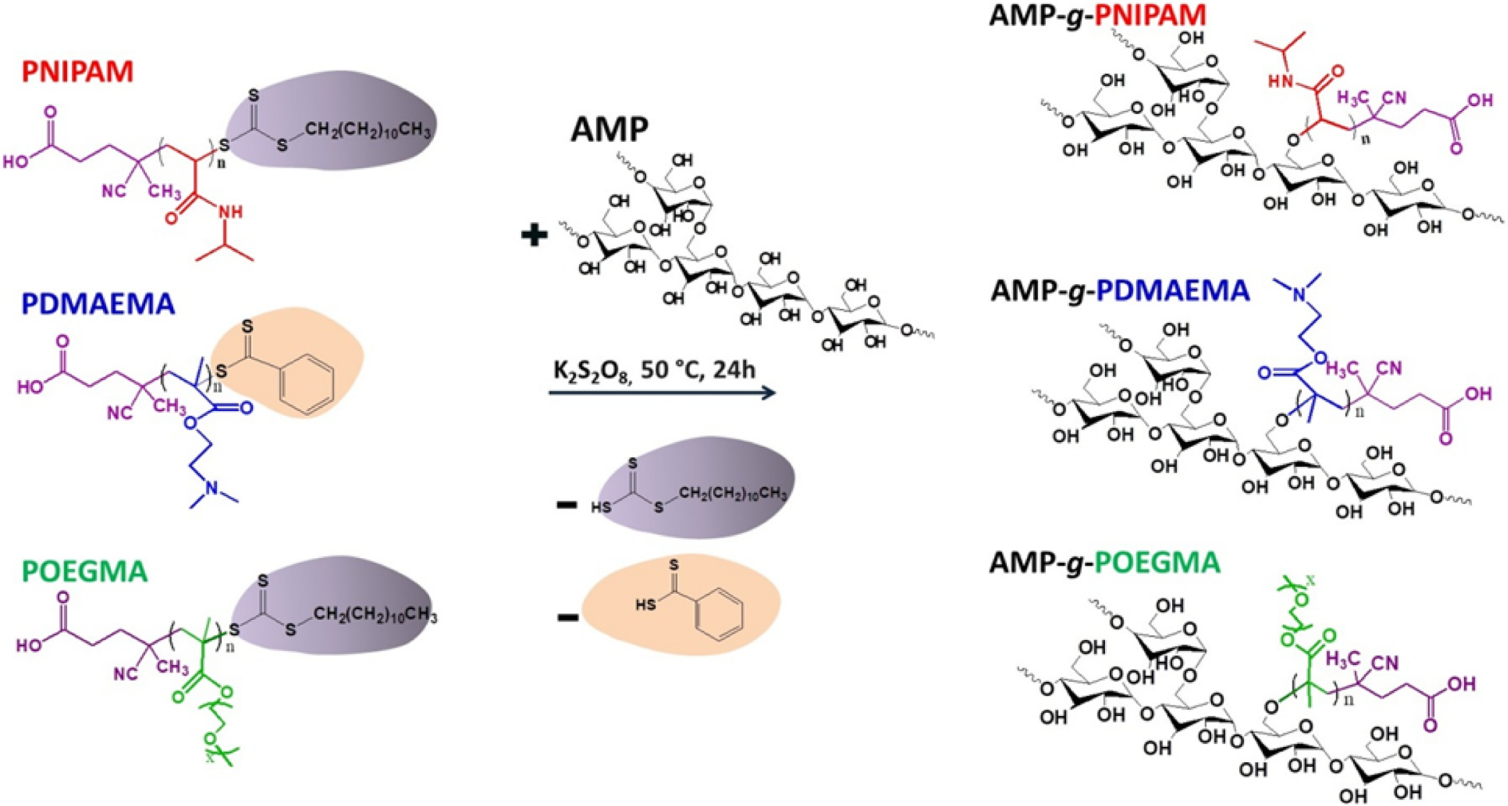
Scheme of the AMP-*g*-PNIPAM, AMP-*g*-PDMAEMA, and AMP-*g*-POEGMA Graft Copolymer Synthesis

### Characterization Methods

FTIR spectroscopy was applied to elucidate the structural characteristics of the freeze-dried copolymers by using the attenuated total reflection (ATR) technique by means of an IR Tracer-100 FTIR spectrometer (Shimadzu Corporation; Kyoto, Japan) having a GladeATR module type (PIKE Technologies; Madison, WI, USA). The spectra were acquired in the 400–4000 cm^–1^ range, with 4 cm^–1^ resolution, at 25 °C.

^1^H NMR spectra were registered using a Bruker Avance NEO 400 MHz spectrometer (Bruker, Rheinstetten, Germany) with a 5 mm QNP direct detection probe and z-gradients. The spectra of copolymer solutions in DMSO-d6 were recorded at 25 °C. The peaks at around 2.512 ppm were assigned to solvent hydrogen atoms, whereas the high peak at 3.4 ppm corresponds to water in the solvent.

The number-average molecular weight (*M*_n_) and dispersity (Đ) of the RAFT-synthesized polymers were determined by performing gel permeation chromatography (GPC) analysis. The GPC measurements were carried out using an OMNISEC multidetector GPC/SEC system (Malvern Panalytical Limited, UK), equipped with a PL-EMD 950 evaporative mass detector (Polymer Laboratories) using two PLgel 5 μm Mixed C and Mixed D Agilent columns. The temperature inside the columns was set at 25 °C, and tetrahydrofuran was used as the solvent at a flow rate of 1 mL·min^–1^. The calibration measurements were performed using polystyrene standards with molecular weights ranging from 580 to 3,150,000 Da.

A thermogravimetric analyzer type Discovery TGA 5500 (TA Instruments, New Castle, USA) was used for analyzing the thermal behavior of amylopectin, synthetic RAFT homopolymers, and the obtained graft copolymers. The samples (approximately 5 mg) were investigated under an inert atmosphere (nitrogen flow: 40 mL·min^–1^) in the temperature domain 37–700 °C, with a heating rate of 10 °C·min^–1^.

Light scattering measurements (dynamic and electrophoretic) were carried out using a Litesizer DLS 500, Anton Paar (Graz, Austria), with a semiconductor laser diode of 40 mW operating at a 658 nm wavelength and a 90° scattering angle. The stimuli-responsiveness of the graft copolymer solutions (0.1 mg mL^–1^) was investigated by measuring the particle size vs temperature (in the range of 25–55 °C), pH (in the range of 4–10, at 25 °C), and ionic strength (varied through consecutive titration with NaCl 1N aqueous solution at 25 °C). The samples were measured after a 60 s equilibration period in the measuring cell at least 3 times, as an average of 3 runs, each of 10 s duration. The zeta potential values of synthetic polymers and the corresponding graft copolymers were studied at different solution pHs and shown as an average of 100 individual runs. Initially, the measurements were performed for the samples prepared in ultrapure water (no pH regulation) and after the pH was decreased to 3 and increased to 10 by adding suitable amounts of HCl and NaOH 0.1 M aqueous solutions, respectively.

## Results and Discussion

### Synthesis and Characterization of the Graft Copolymers

The “grafting to” reaction described in Scheme 1 allows the coupling of reactive radicals produced at the functional end group of the synthetic polymers with the −OH groups of the polysaccharide, leading to covalent bonding between the branched AMP main chain and the PNIPAM, PDMAEMA, and POEGMA linear chains, resulting in the formation of graft copolymers. Nongrafted polymer chains are expected to be removed by the extensive dialysis step performed after the coupling reaction. The ATR-FTIR measurements were performed first to verify the chemical structure of the obtained graft copolymers as compared to the initial components, AMP, and the corresponding synthetic homopolymers. As a common observation for all three copolymers, their spectra contained the main characteristic bands of both AMP and synthetic polymers (PNIPAM, PDMAEMA, and POEGMA) (Figure S4). Thus, the broad band at around the 3600–3200 cm^–1^ region is related to the vibration of the stretching of the AMP backbone −O–H groups and to the −N–H bonds of PNIPAM/PDMAEMA (in the AMP-*g*-PNIPAM/AMP-*g*-PDMAEMA spectra). The absorption bands in the 2975–2870 cm^–1^ region indicate the presence of C–H symmetric/asymmetric vibrations of the CH_2_ and CH_3_ (PNIPAM: −NH–CH–(CH_3_)_2_; PDMAEMA: −CH–N­(CH_3_)_2_; POEGMA: −CH_2_–CH_2_–O–, −CH_3_).^55^ Also, the spectra of the graft copolymers present strong absorption bands characteristic of AMP in the 1800–850 cm^–1^ fingerprint region (Figure 1), mainly at around 1150, 1080, and 1010 cm^–1^, due to the vibration of −C–O–C linkages and −C–O–H groups of the anhydroglucosidic rings of AMP.^5^

**Figure 1:**
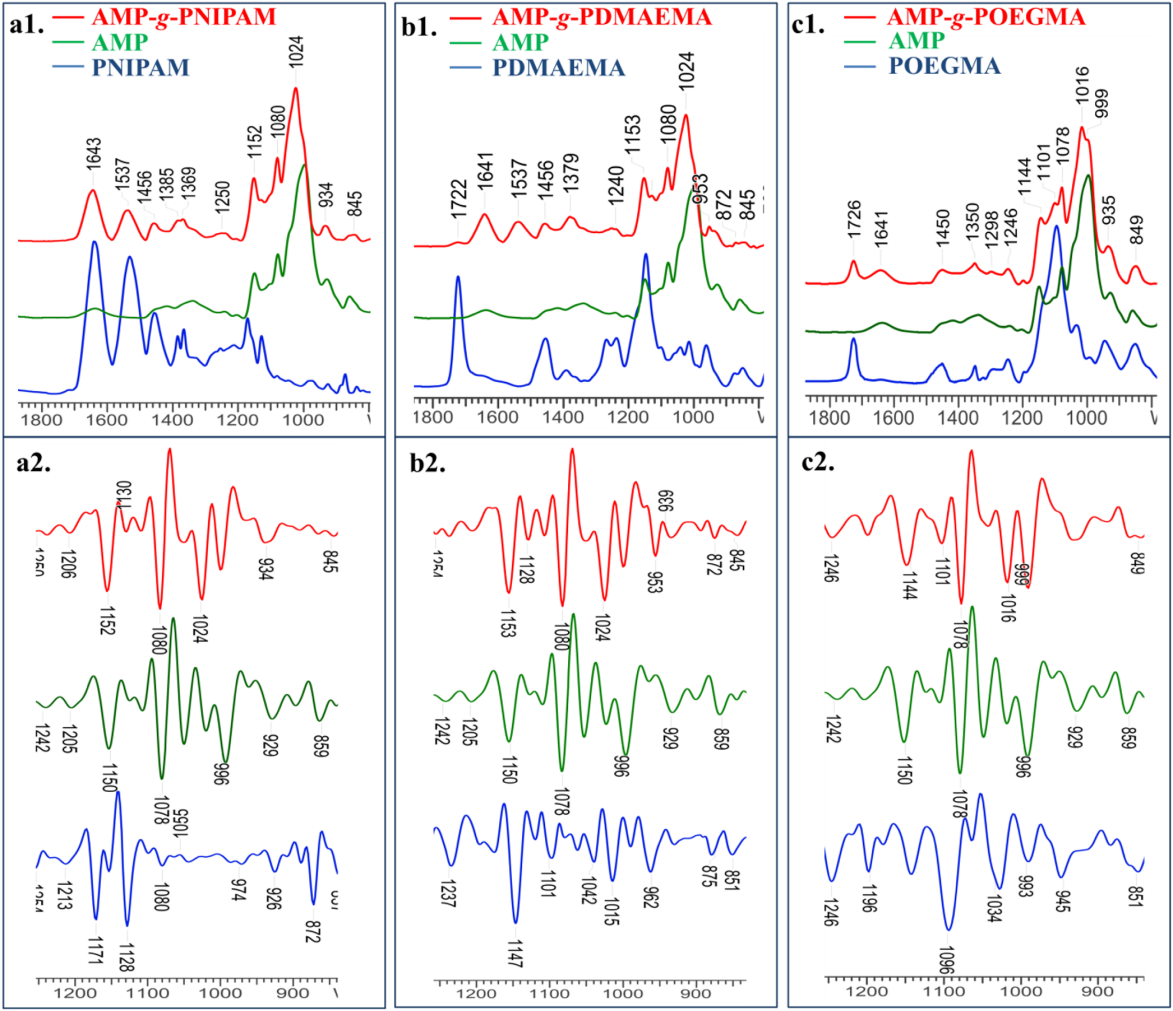
ATR-FTIR (a1, b1, c1) and second derivative (a2, b2, c2) spectra of synthetic polymers and AMP and graft copolymers (AMP spectra were introduced in all figures for comparison).

The characteristic bands of PNIPAM are presented in the infrared spectrum of the AMP-*g*-PNIPAM copolymer (Figure 1, a1) at 1640 cm^–1^ assigned to the carbonyl (C=O) stretching of amide I and at 1535 cm^–1^ both to −C–N stretching and −N–H bond deformation of amide II, respectively. The PNIPAM characteristic bands appear at 1458 cm^–1^ and 1365 cm^–1^ corresponding to C–H stretching (−CH_2_ bending and isopropyl group (−CH–(CH_3_)_2_).^64^ Moreover, in the AMP-*g*-PNIPAM spectrum, a shift from 996 cm^–1^ to 1024 cm^–1^ in the stretching vibration can be observed, the signal being specific to C–O bonds of amylopectin, which suggests the reaction of −OH groups of AMP with the synthetic polymer and the increased content of aliphatic ether linkages. This assumption was also confirmed by comparing the PNIPAM, AMP, and AMP-*g*-PNIPAM second derivative spectra (Figure 1a2), where in the range 990–1080 cm^–1^ an increase of the absorbance was observed for the graft copolymer as compared to neat AMP. Moreover, the decrease in the 860 cm^–1^ band intensity, specific to C–OH bonds in AMP, further supports the grafting of AMP with PNIPAM. Another indication of the realization of the grafting reaction, obtained from the FTIR spectra, is the absence of the band at 872 cm^–1^ in the graft copolymer second derivative spectrum, characteristic of the C–S end-chain bonds of the fragment remaining from the chain transfer agent used in the NIPAM polymerization by RAFT, which supports the PNIPAM fragmentation during the grafting reaction.

The ATR-FTIR spectrum of the AMP-*g*-PDMAEMA copolymer (Figure 1b1) presents characteristic bands of PDMAEMA at 1722 cm^–1^, 1641 cm^–1^, and 1535 cm^–1^ attributed to the stretching of the C=O and −C–O groups and to the deformational stretching of −N­(CH_3_)_2_, respectively. Bands appearing at 1460 cm^–1^ and 1379 cm^–1^ are assigned to the bending of the −CH_2_ and −CH_3_ groups.^5^ Also, in the spectrum of AMP-*g*-PDMAEMA, the increase in the content of −C–O–C– linkages can be observed, as in the AMP-*g*-PNIPAM case, through the shift of the signal attributed to the −C–O bonds from 996 cm^–1^ to 1024 cm^–1^ denoting the reaction of the −OH groups of AMP with the RAFT-obtained synthetic homopolymer.^56^ This statement is in agreement with the results obtained from the second derivative spectra of PDMAEMA, AMP, and AMP-*g*-PDMAEMA (Figure 1, b2). Thus, at 1024 cm^–1^, there is an increase of intensity for the signal assigned to −C–O–C– ether bond vibrations, thus supporting the successful formation of new aliphatic ether bonds. Besides, in the spectrum of the graft copolymer, a typical absorption band of the C–O–C group can be found at 953 cm^–1^, characteristic of the groups O=C—O—C of the synthetic polymer, which suggests that grafting of PDMAEMA onto amylopectin took place.^57,58^

In the spectrum of the AMP-*g*-POEGMA copolymer (Figure 1c1), the bands at 1721 cm^–1^ and 1015 cm^–1^ can be allocated to the —C=O and −C–O carbonyl vibrations of the ester group. The new signal detected at 1101 cm^–1^ is related to the −C–O–C– stretch of the −OCH_2_CH_2_ side chain repeating units of POEGMA, while the bands characteristic of −CH, −CH_2_, and −CH_3_ bonds’ stretching vibrations are at 1450 cm^–1^, 1350 cm^–1^, and 1298 cm^–1^, respectively.^59,60^ Furthermore, from the POEGMA, AMP, and AMP-*g*-POEGMA second derivative spectra (Figure 1c2), the grafting of POEGMA onto amylopectin could be established by the intensity increase of the alkyl ether (−C–O–C−) stretch signal at 1015 cm^–1^.

Figure 2 summarizes the ^1^H NMR spectra of AMP and the obtained grafted copolymers. The ^1^H NMR spectrum of the AMP-*g*-PNIPAM graft copolymer displays peaks corresponding to both the AMP and the PNIPAM structures, confirming the successful grafting of PNIPAM onto the AMP backbone. Peaks observed in the 3.4–3.6 ppm range are attributed to protons from the carbon atoms at positions C2, C3, C4, C5, and C6 of the anhydroglucosidic unit of AMP. The peaks at 5.11 and 4.91 ppm are attributed to protons from the carbon atoms in the C1 and C1’ positions, corresponding to α-(1,4) and α-(1,6) glycosidic bonds between the d-glucopyranose units of AMP. Additionally, the peaks at 4.52, 5.45, and 5.51 ppm are assigned to protons from the hydroxyl groups of amylopectin at the HO-C6, HO-C2, and HO-C3 positions, respectively. The characteristic peaks of the synthetic polymer PNIPAM are located at 1.05 ppm, assigned to protons from methyl groups −CH_3_ (C11), and 1.45 and 1.98 ppm, assigned to protons from groups −CH_2_ (C8) and −CH (C7), respectively.

**Figure 2:**
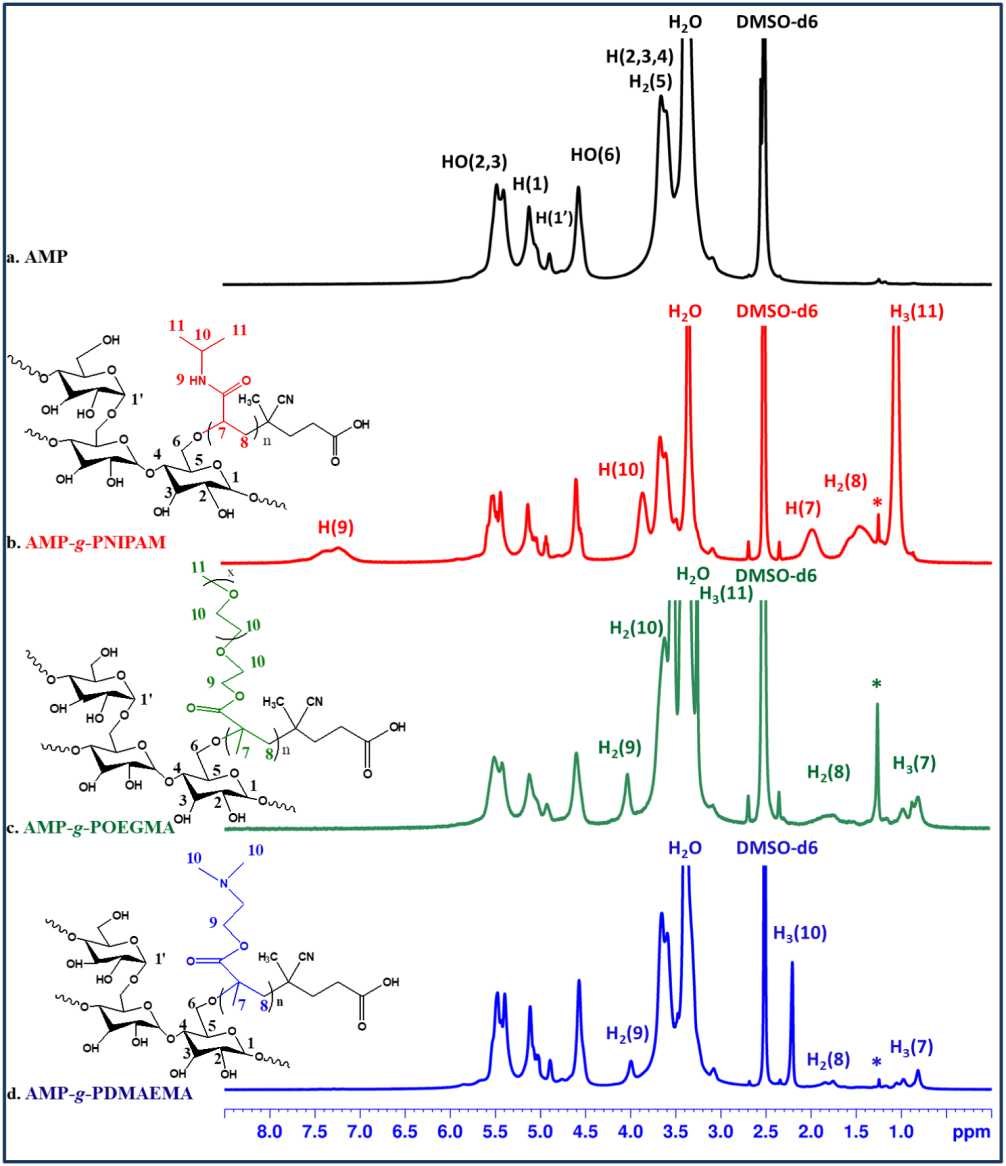
^1^H NMR spectra of AMP (a), AMP-*g*-PNIPAM (b), AMP-*g*-POEGMA (c), and AMP-*g*-PDMAEMA (d) (*-*n*-hexane).

The signals observed in the ^1^H NMR spectrum of AMP-*g*-POEGMA in the range 3.37–3.61 ppm are associated with the overlapping of proton signals from the carbon atoms of the amylopectin unit (−CH–, positions C2–C6) to the −CH_2_– protons characteristic of the POEGMA repeating unit and the −O–CH_2_–CH_2_– moiety (C10). The specific signals corresponding to the protons of the —CH_2_—O—C=O— ester bond (C9) and the −CH_3_ groups (C11) of the synthetic polymer appear at 4.02 and 3.25 ppm, respectively. In addition, the signals associated with the −CH_3_ (C7) and −CH_2_ (C8) groups are observed in the regions 0.8–1.2 and 1.7 ppm, respectively. Furthermore, in the spectrum of the AMP-*g*-POEGMA copolymer, characteristic peaks at 4.49, 5.40, and 5.50 ppm correspond to the protons of hydroxyl groups attached to the carbon atoms at positions C6, C2, and C3, respectively.

For the AMP-*g*-PDMAEMA copolymer, the characteristic peaks of the protons in the −CH_3_ (C7) and −CH_2_– (C8) groups of PDMAEMA appear at 1.80 and 0.89 ppm, respectively. Additionally, peaks observed at 2.2 and 4.0 ppm correspond to the protons of −CH_3_ from the −N­(CH_3_)_2_ moiety (6H, C10) and to the protons of the −CH_2_– group (C9), respectively. The signals associated with the hydroxyl protons at positions C6, C2, and C3 are located at 4.6, 5.4, and 5.5 ppm, respectively. The absence of signals in the 7.4–8 ppm range—typically attributed to aromatic protons of the synthetic polymer—suggests the cleavage of the aromatic fragment from the POEGMA homopolymer chain end and supports the successful grafting of the synthetic polymer onto the amylopectin backbone.

The degrees of substitution (DOS) for all three new copolymers were calculated using eq 1:^61^

1 $$\mathrm{DOS}=\left(\frac{I_{1,05/6}}{I_{5,11}}\right):3$$

Thus, the DOS values were determined from the ratio between the integral of the methyl proton signal at 1.05 ppm (H11/6) from PNIPAM, the H7/3 proton signal at 0.85 ppm from POEGMA, or the methyl proton signal at 2.20 ppm (H10/6) from PDMAEMA to the anomeric proton signal at 5.11 ppm (H1) from AMP, resulting in values of 0.27, 0.07, and 0.03 for copolymers AMP-*g*-PNIPAM, AMP-*g*-PDMAEMA, and AMP-*g*-POEGMA, respectively. The results sustain the partial grafting observed by ATR-FTIR spectroscopy, as not all reactive sites on the AMP backbone participated in the reaction due to steric hindrances that appear as a result of the high molar mass and branched structure of AMP. Nevertheless, the DOS value can be correlated with sufficient grafting efficiency to ensure stimuli-responsive properties to the so-obtained copolymer. Moreover, the lower DOS values, indicating a low level of grafting, were obtained when POEGMA and PDMAEMA were used, considering that these synthetic polymers have a bulkier structure as compared to PNIPAM, and therefore, steric hindrances occur. Thus, the resulting copolymers are predominantly composed of AMP, grafted with synthetic polymer side chains.

The thermal stability of graft copolymers as compared to that of the starting materials was investigated by thermogravimetric analysis, with the obtained thermograms being presented in Figure 3. In the investigated thermogravimetric curves, a weight loss around 70 °C can be observed, attributed to the residual solvent in the analyzed samples and the evaporation of absorbed water. The thermal degradation of the AMP-*g*-PNIPAM copolymer occurs in two stages (Figure 3a). The first stage, in the 280–360 °C temperature range, with a maximum at 323 °C, indicates the thermal decomposition of amylopectin (approximately 50% weight loss). The second stage of thermal degradation, which started at a temperature above 370 °C and continued up to 440 °C, is related to thermal degradation of the PNIPAM synthetic polymer, with about 25% weight loss. By comparing the thermograms of the AMP and PNIPAM with that of the AMP-*g*-PNIPAM copolymer, an improvement in the thermal stability of the obtained copolymer can be noticed, with the weight loss in the first degradation step decreasing from 70%, in the case of AMP, to ∼50% for AMP-*g*-PNIPAM.

**Figure 3:**
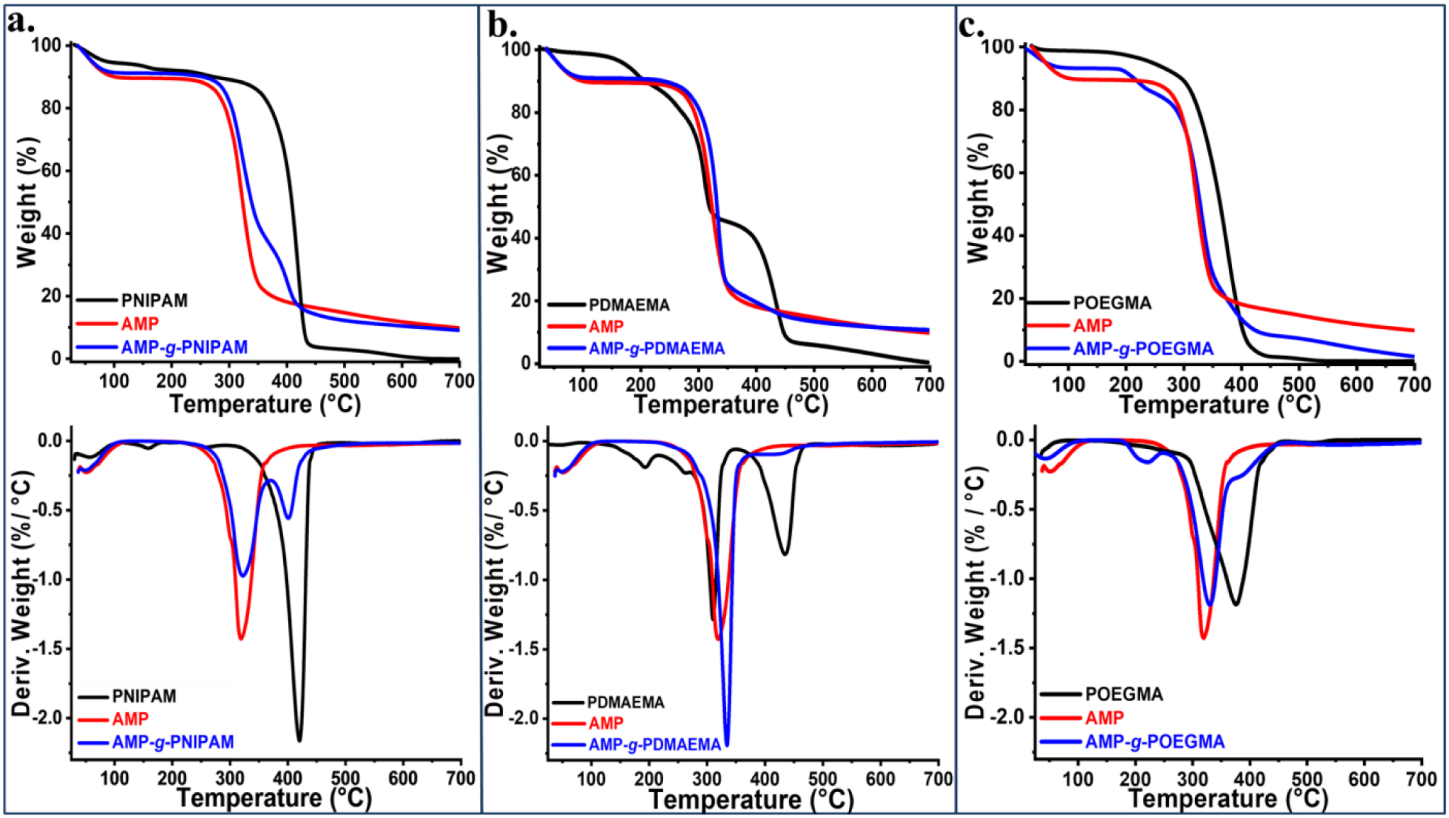
TGA and DTG thermograms of synthetic polymers, AMP, and graft copolymers (AMP-*g*-PNIPAM (a), AMP-*g*-PDMAEMA (b), and AMP-*g*-POEGMA (c)). AMP curves are represented in all thermograms for comparison.

TGA/DTG thermograms of PDMAEMA and AMP-*g*-PDMAEMA are presented in Figure 3b. Thus, at around 130 °C, the PDMAEMA homopolymer starts to thermally degrade, showing three degradation steps: the first step in the temperature range of 130–280 °C (approximately 10% weight loss), the second in the range of 280–340 °C (approximately 45% weight loss), and the third in the range between 370 and 460 °C (approximately 40% weight loss). The thermal degradation of the AMP-*g*-PDMAEMA copolymer occurred in two stages between 270 and 460 °C. The first stage of thermal degradation is the major one (270–350 °C), with about 70% weight loss, which is probably induced by the simultaneous partial degradation of PDMAEMA and the decomposition of amylopectin. The second stage, relatively smaller (∼5% weight loss), can be observed in the 400–500 °C temperature range and is specific to the degradation of the methacrylate-grafted homopolymer. The thermal stability of the AMP-*g*-POEGMA graft copolymer was also estimated as compared to the thermal behavior of the initial components, AMP and POEGMA (Figure 3c). Thus, POEGMA shows better thermal stability than AMP in the range of 100–400 °C, followed by its complete decomposition at ca. 450 °C. In the case of the AMP-*g*-POEGMA copolymer, the thermal degradation curves revealed that the degradation starts at ca. 250 °C, with a weight loss of about 10%. The principal thermal decomposition step started at 300 °C with a maximum at about 320 °C (approximately 70% weight loss) and corresponds mainly to the degradation of AMP since a further decomposition started at 380 °C corresponding to the side chain degradation of the graft copolymer, resulting in complete thermal decomposition by ca. 450 °C. In summary, the hybrid copolymer thermal stability is dictated mainly by the AMP as the main component in the chemical structure of the copolymers and can be increased by the presence of the synthetic polymer. Nevertheless, the low content of synthetic polymer grafted to AMP is reflected by less influence on the overall thermal behavior of the copolymers.

### Graft Copolymer Behavior in Aqueous Solutions

Since AMP is not soluble in water, while the grafted homopolymers are water-soluble, the obtained graft copolymers are expected to show amphiphilicity and some degree of aggregate formation in aqueous media. Moreover, taking into account the functional groups of the synthetic polymers, the aggregation behavior is expected to vary according to the solution’s pH. Figure 4 shows the zeta potential variation function of the pH of the solutions of the obtained copolymers and of the corresponding synthetic polymers. The PNIPAM and AMP-*g*-PNIPAM copolymer (Figure 4a) show a negative zeta potential over almost the entire range of investigated solution pH, decreasing with the pH increase. As pH-sensitive groups, PNIPAM contains carboxyl end groups, which are deprotonated in the studied pH range. The AMP-*g*-PNIPAM copolymer is also negatively charged along the investigated pH range and exhibited an “isoelectric point” (zero zeta potential) at pH 3, similar to that of PNIPAM, denoting that the zeta potential values of the copolymer are strictly connected to the deprotonation of PNIPAM carboxylic end group.

**Figure 4:**
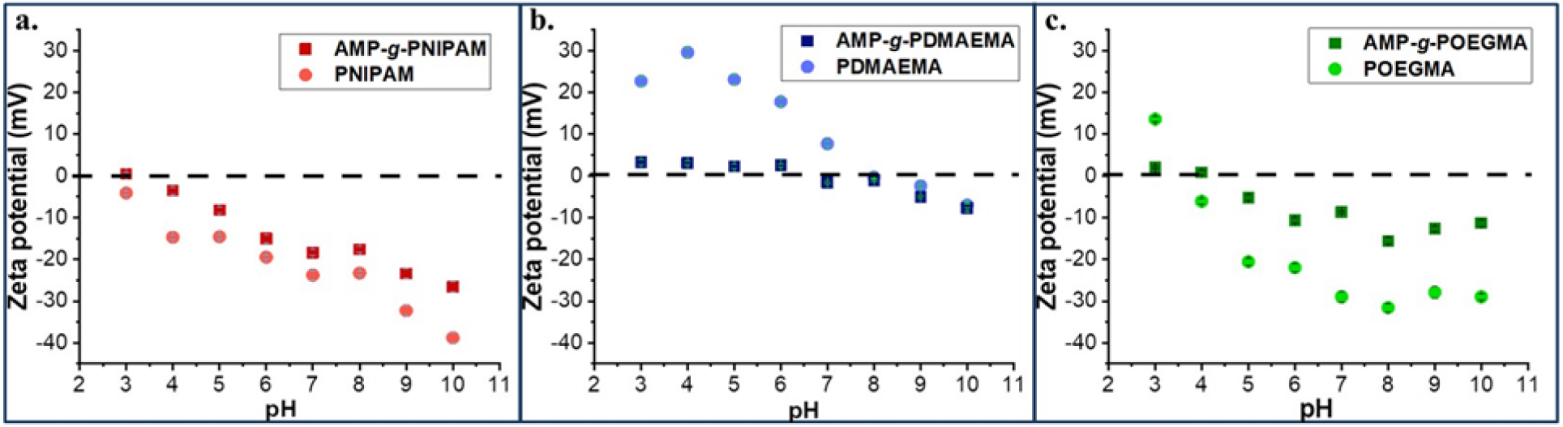
Zeta potential values vs pH of (a) AMP-*g*-PNIPAM and PNIPAM, (b) AMP-*g*-PDMAEMA and PDMAEMA, and (c) AMP-*g*-POEGMA and POEGMA (standard deviation of maximum 1.4 mV).

In the case of PDMAEMA and AMP-*g*-PDMAEMA (Figure 4b), a variation of the zeta potential values as a function of pH, from positive to negative values, can be observed. PDMAEMA has an isoelectric point at a pH of about 8, being positively charged under physiological conditions. The positive surface charge of PDMAEMA in an acidic environment arises from the tertiary amine group protonation, while in basic conditions, the deprotonation of carboxylic end groups occurs, resulting in an apparent zeta potential with negative values. Similar to the synthetic polymer, the apparent zeta potential values of the AMP-*g*-PDMAEMA copolymer in the pH range of 3–7 are slightly positive, with the isoelectric point being located at a pH of about 7, confirming the presence of PDMAEMA grafts but also the low grafting degree on the copolymer. In the case of POEGMA and AMP-*g*-POEGMA copolymers (Figure 4c), the values of zeta potential are negative over almost the entire range of tested pH, and they can also be associated with the negatively charged carboxyl groups attached to the ends of POEGMA grafted chains, similar to the case of the PNIPAM-based graft copolymer.

Following the variation of the zeta potential vs pH, the variation of the formed aggregate size was consequently followed by DLS, with the corresponding size distributions being presented in Figure 5, whereas the measured scattered intensity and the hydrodynamic diameter are presented in Table 1.

**Figure 5:**
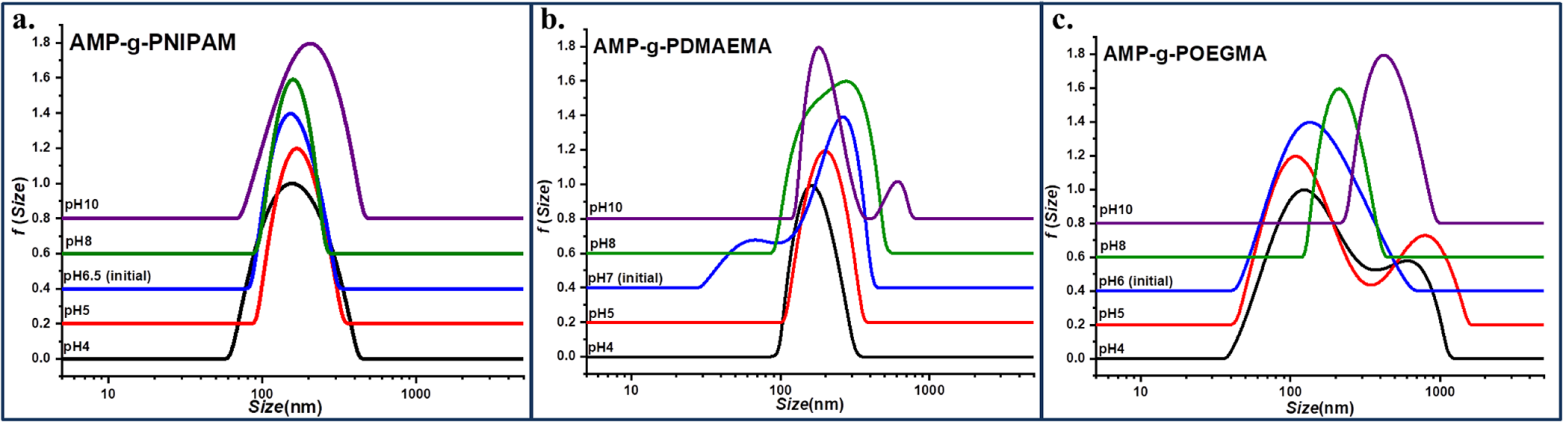
Size distribution as a function of pH for (a) AMP-*g*-PNIPAM, (b)­AMP-*g*-PDMAEMA, and (c) AMP-*g*-POEGMA.

**Table 1:** Light Scattered Intensity (*I*) and Hydrodynamic Diameter (Dh) of Graft Copolymers in Aqueous Media of Various pH

	AMP-*g*-PNIPAM		AMP-*g*-PDMAEMA		AMP-*g*-POEGMA	
pH	*I* (kcps)	Dh (nm)	*I* (kcps)	Dh (nm)	*I* (kcps)	Dh (nm)
4	876	181	1957	215	556	362
5	852	207	1844	230	491	336
Initial pH (6.5/7/6)	985	174	887	264	822	238
8	670	214	401	303	305	325
10	686	270	581	433	588	410

The size distributions from DLS measurements for the AMP-*g*-PNIPAM graft copolymer indicate the formation of nearly monodisperse aggregates over the entire pH range (Figure 5a). At the whole investigated pH range, the copolymer is rather hydrophilic due to the hydrophilic NIPAM segments and the deprotonated terminal carboxyl groups of PNIPAM (Figure 4a), and therefore, the variation of size distributions is quite small. The deprotonation of carboxyl groups induces electrostatic repulsions among these negatively charged terminal groups on the PNIPAM-grafted chains, leading to extended copolymer chains, and therefore, the found particle sizes are relatively high. Also, the higher values of light-scattered intensity for the initial solution (Table 1) as compared to the ones with pH adjusted, either to acidic or basic pH, show that the added counterions partially hindered the ionic charges on the copolymer and led to looser and less dense particles in the aqueous solution, with a hydrodynamic diameter in the range of 180–270 nm.

As a weak cationic polyelectrolyte, the ionization degree of PDMAEMA side chains depends on pH. Therefore, in the case of the AMP-*g*-PDMAEMA copolymer, the change in ionization (Figure 4b), even low, is expected to induce conformational changes of the macromolecule. As shown in Figure 5b, at the pH of ultrapure water (pH 7), which is close to the p*K*_a_ value of AMP-*g*-PDMAEMA (Figure 4b), a high tendency of aggregation and a high size distribution were found. The changes in pH affected both the scattered intensity and the hydrodynamic diameter of the aggregates (Table 1). Thus, by changing the pH from 7 to 4, both a reduction in the hydrodynamic diameter and an increase in scattered intensity are evident, suggesting the contraction of chains and the formation of denser aggregates. Under acidic conditions (pH 4), the observed decrease in particle size to 215 nm can be attributed to the shrinking of the copolymer particles and the rearrangements of the supramolecular structures formed at pH 7, which result from the tertiary amine groups’ partial protonation and the presence of weak electrostatic repulsion. Also, the hydrodynamic diameter increase at 433 nm under alkaline conditions (pH 10) is related to the aggregation of the macromolecular chains, whereas the tertiary amine groups are practically uncharged and the electrostatic repulsion between the carboxylic end groups of the RAFT-obtained PDMAEMA chains is low since the grafting density is just about 7%, as found by ^1^H NMR.

At the initial pH (pH 6) and below it, the copolymer AMP-*g*-POEGMA exhibits a bimodal size distribution (Figure 5c), attributed to the partially ionized carboxylic acid groups of POEGMA (Figure 4c). With the increase of pH to 10, the terminal −COOH groups become deprotonated, leading to an intensification of the electrostatic repulsions between the negatively charged groups, and thus, a monomodal size distribution is observed, together with a pronounced effect on the hydrodynamic diameter values, increasing from 239 to 410 nm (Table 1).

It is well-known that starch gelatinization (temperature-induced gelatinization) exhibits different behavior depending on the amylose/amylopectin ratio, which varies according to the starch source (maize, cassava, wheat, and potato). Having different molecular architectures and physicochemical behaviors, amylose and amylopectin interact differently in water and under thermal treatment. Depending on the amylose/amylopectin ratio, the gelatinization occurs within a temperature range of 60–80 °C, with higher temperature gelatinization also being found for starch with a higher amount of amylopectin, at around 73 °C.^64,62,63^ Since the grafted copolymers were obtained using three thermoresponsive polymers, which exhibit different LCST, and for a better understanding of their behavior and stability upon temperature variation, the DLS measurements in terms of size distribution, hydrodynamic diameter, and scattered intensity were also investigated as a function of temperature (Figure 6). The temperature range was established according to each polymer’s LCST, as known from the literature.

**Figure 6:**
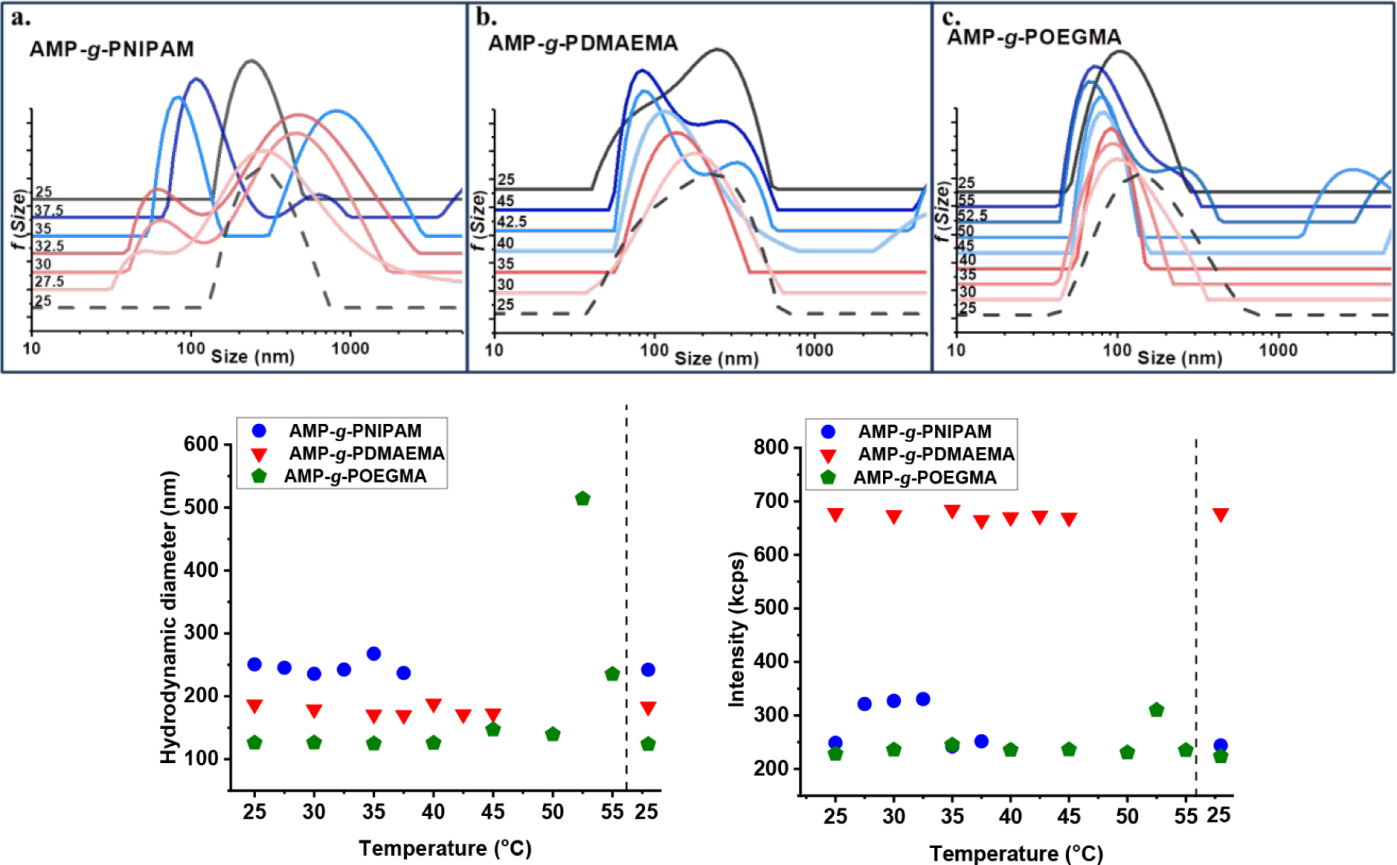
Size distribution, hydrodynamic diameter, and scattered intensity as a function of temperature for (a) AMP-*g*-PNIPAM, (b) AMP-*g*-PDMAEMA, and (c) AMP-*g*-POEGMA copolymer aqueous solutions.

As shown in Figure 6a, DLS measurements of the AMP-*g*-PNIPAM copolymer were carried out in the temperature range of 25–37.5 °C, followed by cooling back to 25 °C. Due to the strong intermolecular hydrogen bonding interactions between its backbones, AMP is insoluble in water. The grafting of PNIPAM, which contains hydrophilic side groups, onto the AMP backbones is expected to change the structural regularity of AMP, leading to a weakening of polysaccharide backbone interactions. With the increase in temperature, the copolymer starts to exhibit a bimodal size distribution of the aggregates, ranging from approximately 30 to 1000 nm. This behavior may be attributed to the swelling of AMP as a result of the presence of PNIPAM branches. Wang et al.^39^ studied the thermal response of the starch-*g*-PNIPAM copolymer and demonstrated that the LCST decreases from 31.5 to 23 °C, depending on the length of PNIPAM chains. Accordingly, for the AMP-*g*-PNIPAM copolymer investigated in this study, the LCST value is located right above 25 °C, with large size polydispersity being observed for all the investigated range of temperatures (27.5–37.5 °C), suggesting that the hydrophobic groups are fully exposed with an increase in interchain interactions and the formation of hydrophobic aggregates of different sizes. After being cooled to 25 °C, the AMP-*g*-PNIPAM copolymer returned to its initial particle size distribution, indicating the reversibility of the process. The particle size distribution, hydrodynamic diameter, and scattered intensity from DLS measurements of the AMP-*g*-PDMAEMA graft copolymer were studied by a heating/cooling protocol in the temperature range of 25–45 °C and cooling back to 25 °C. In the pH aqueous media, PDMAEMA is expected to exhibit an LCST in the range of 40–50 °C.^43^ For the AMP-*g*-PDMAEMA copolymer, the measured pH in ultrapure water was 7.4, which is close to the PDMAEMA p*K*_a_ value.^44^ The DLS results showed a quite small variation in the hydrodynamic diameter within the 25–40 °C temperature range during heating and indicate the formation of monodisperse aggregates. As the temperature increases above 40 °C, a bimodal size distribution of the AMP-*g*-PDMAEMA aggregates was observed, indicating the LCST value for this copolymer. The increase in intensity of the peak in the 50–100 nm range indicated that the number of aggregates increased, caused by the increase in hydrophobic interactions that promote aggregation. Similarly to the AMP-*g*-PNIPAM copolymer, after cooling to 25 °C, AMP-*g*-PDMAEMA returned to its initial self-assembled state. Since POEGMA has an LCST around 50 °C, DLS measurements of the AMP-*g*-POEGMA copolymer in terms of particle size distribution, hydrodynamic diameter, and scattered intensity were performed in the 25–55 °C temperature range, followed by cooling back to 25 °C. The monodisperse aggregates with a narrow particle size distribution observed during the heating of AMP-*g*-POEGMA from 25 to 50 °C indicate that no significant changes in particle size occurred with the increase of temperature up to 50 °C. Besides, this assumption is supported by the hydrodynamic diameter and scattered intensity values obtained during the same temperature range. Above 50 °C, DLS results revealed a bimodal size distribution of the aggregates with an increase of hydrodynamic diameter and scattered intensity values, suggesting that, close to the POEGMA LCST, the copolymer experiences increased hydrophobic interactions, promoting the formation of multichain aggregates. After cooling back to 25 °C, the AMP-*g*-POEGMA copolymer exhibited behavior similar to that observed for the other two copolymers under study, also indicating the reversibility of the observed transitions in the system.

The effect of increasing ionic strength on the grafted copolymer solutions represents another important external factor that needs to be investigated since PNIPAM, PDMAEMA, and POEGMA are known to exhibit ionic strength responsive behavior. As shown in Figure 7, DLS measurements, in terms of particle size distribution, hydrodynamic diameter, and scattered intensity, were performed to detect the response of the copolymer aggregates to salt addition. For the AMP-*g*-PNIPAM graft copolymer (Figure 7a), a decrease in the sizes of the aggregates was observed by the addition of NaCl. The conformational changes indicate a decrease in the repulsive electrostatic interactions between copolymer chains and the formation of smaller, more hydrophobic aggregates, leading to a more collapsed conformation with increasing salt concentration. Also, the AMP-*g*-PDMAEMA (Figure 7b) and AMP-*g*-POEGMA (Figure 7c) graft copolymers exhibit similar behavior with an increase of the ionic strength of the copolymer aqueous solution, with an increase in the sizes of the aggregates being observed from the first additions of salt. This behavior suggests a significant reduction in the electrostatic repulsion between copolymer chains from the beginning, promoting the formation of multichain aggregates.

**Figure 7:**
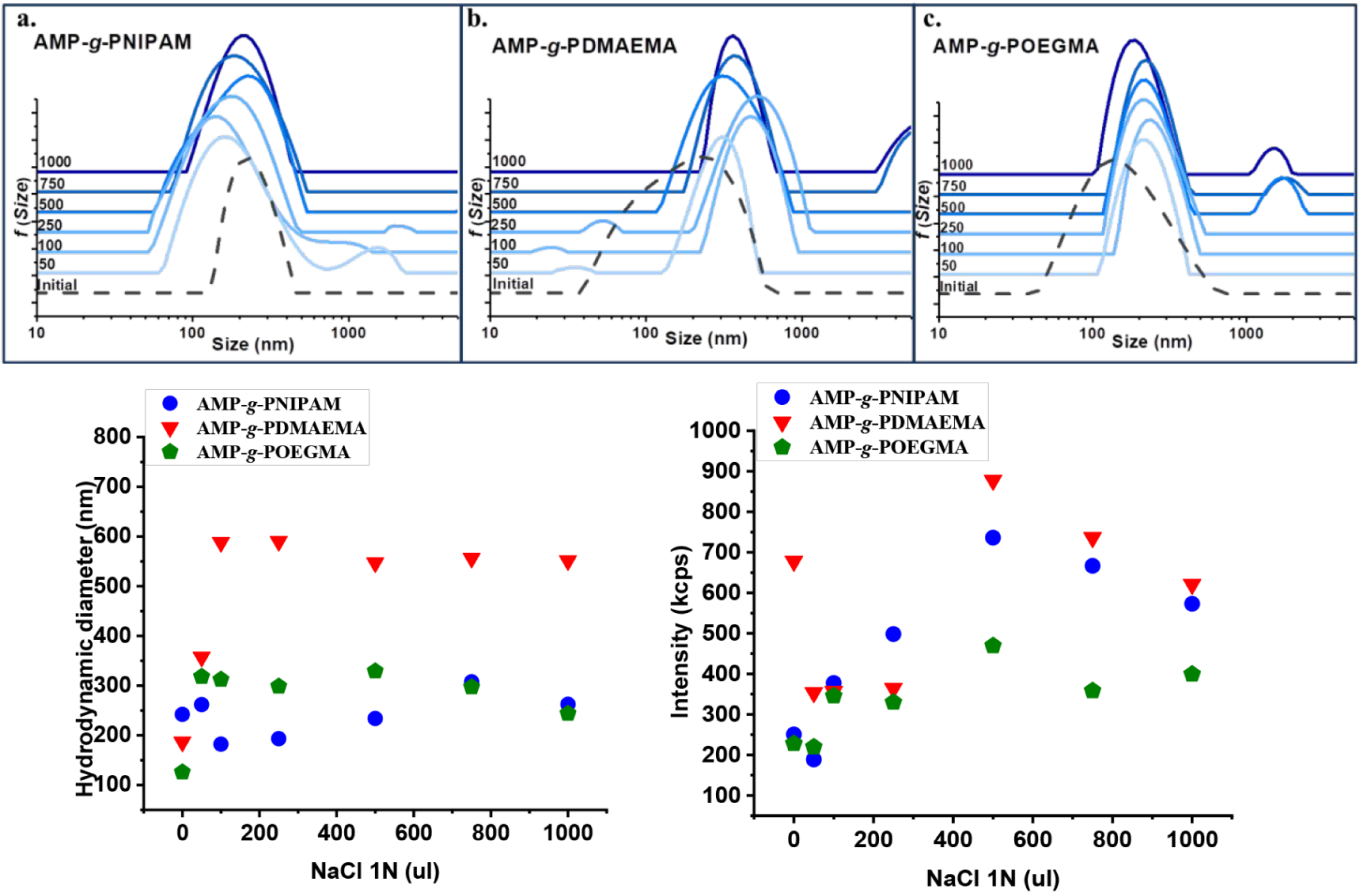
Size distribution, hydrodynamic diameter, and scattered intensity as a function of NaCl concentration for (a) AMP-*g*-PNIPAM, (b)­AMP-*g*-PDMAEMA, and (c) AMP-*g*-POEGMA copolymers.

Cumulatively, these results support the stimuli-responsiveness of the obtained copolymers, with the observed modifications in properties produced by pH, temperature, or ionic strength variations being strongly dependent on the nature of the synthetic polymers grafted on the AMP backbone. The tunable properties observed for these copolymers are particularly attractive in the development of several possible applications, as factors such as pH or temperature control might enhance the functionality and applicability of the copolymers. In this light, the AMP-based copolymers could potentially be tested for applications like smart hydrogels, biosensing, or other systems capable of responding to the mentioned stimuli. However, extensive studies are needed; therefore, further research will be dedicated to assessing the potential applications of the AMP graft copolymers.

## Conclusions

A new “grafting to” strategy for amylopectin has been proposed as an effective method to incorporate designed and tunable stimuli-responsive synthetic polymer chains through RAFT polymerization, forming AMP-*g*-PNIPAM, AMP-*g*-PDMAEMA, and AMP-*g*-POEGMA copolymers and resulting in three types of hybrid graft copolymer materials with pH- and temperature-responsive properties. The strategy provides a method to easily attach different RAFT-synthesized polymers to the branched amylopectin backbone. The AMP-grafted copolymers created in this study exhibit greater advantages over previously published starch-based grafted copolymers due to the unique structural features of amylopectin, as it produces highly branched and defined locations for the attachment of the grafted polymer chains. The RAFT-based synthetic method also allows for an accurate means of controlling the length and proportions of the grafted polymers. The resulting “grafting to” method allows for the creation of grafted polymers with custom tunable grafting densities and a wide variety of behaviors in solution, including thermoresponsiveness and/or pH-responsiveness, which are elucidated in detail in this study. These defining features provide advantages to the AMP-grafted copolymers in comparison with the earlier reported starch-based graft copolymers. Furthermore, this highlights their great potential in the development of advanced functional polymer materials. The successful production of ether linkages between the synthetic and natural polymers was confirmed through spectroscopic techniques (ATR-FTIR and ^1^H NMR). Additionally, this new process to produce hybrid biomacromolecules retains the natural benefits associated with amylopectin while also incorporating a defined set of stimuli-responsive behaviors associated with the synthetic side chains of the grafted product. The hybrid graft copolymers demonstrated significant pH-, temperature-, and ionic strength-triggered self-assembly behaviors in aqueous environments. The chemical composition and ionic properties of the grafted polymers are responsible for these behaviors. Changes in temperature resulted in significant changes in the aggregation behavior of the hybrid graft copolymers produced. Utilizing the “grafting to” methodology, this study has established a versatile yet strong platform for obtaining hybrid systems based on amylopectins that can respond to physical stimuli. In addition, the method developed could provide many opportunities for designing polysaccharide-based advanced systems for smart coatings, nanostructured materials, and bioderived delivery vehicles, which require environmental and interference control in how they assemble and respond to stimuli.

## Supplementary Material



## Notes

Gel permeation chromatography (GPC) results of synthetic polymers (Figure S1); ATR-FTIR spectra of the synthetic polymers (Figure S2); ^1^H NMR spectra of synthetic polymers used for the “grafting to” reactions (Figure S3); and ATR-FTIR spectra of synthetic polymers, AMP, and graft copolymers (Figure S4) (PDF)
