# Supplementary material for: Amylopectin Copolymers Grafted with RAFT-Obtained Synthetic Polymers: Synthesis and Aqueous Solution Behavior
Source: Biomacromolecules. 2026 Feb 10;27(3):1985–98. doi: 10.1021/acs.biomac.5c02198 (PMC12977030; doi:10.1021/acs.biomac.5c02198)
Supplement: Supplementary file 1 [file bm5c02198_si_001.pdf]

Supplementary Material

Amylopectin copolymers grafted with RAFT obtained synthetic polymers. Synthesis and aqueous solution behavior

Melinda-Maria BAZARGHIDEANU, Marius-Mihai ZAHARIA, Ana-Maria MACSIM, Marcela MIHAI, Stergios PISPAS**

Petru Poni Institute of Macromolecular Chemistry, 41A Grigore Ghica Voda Alley, 700487 Iasi, Romania.

*Corresponding authors: marcela.mihai@icmpp.ro, pispas@eie.gr

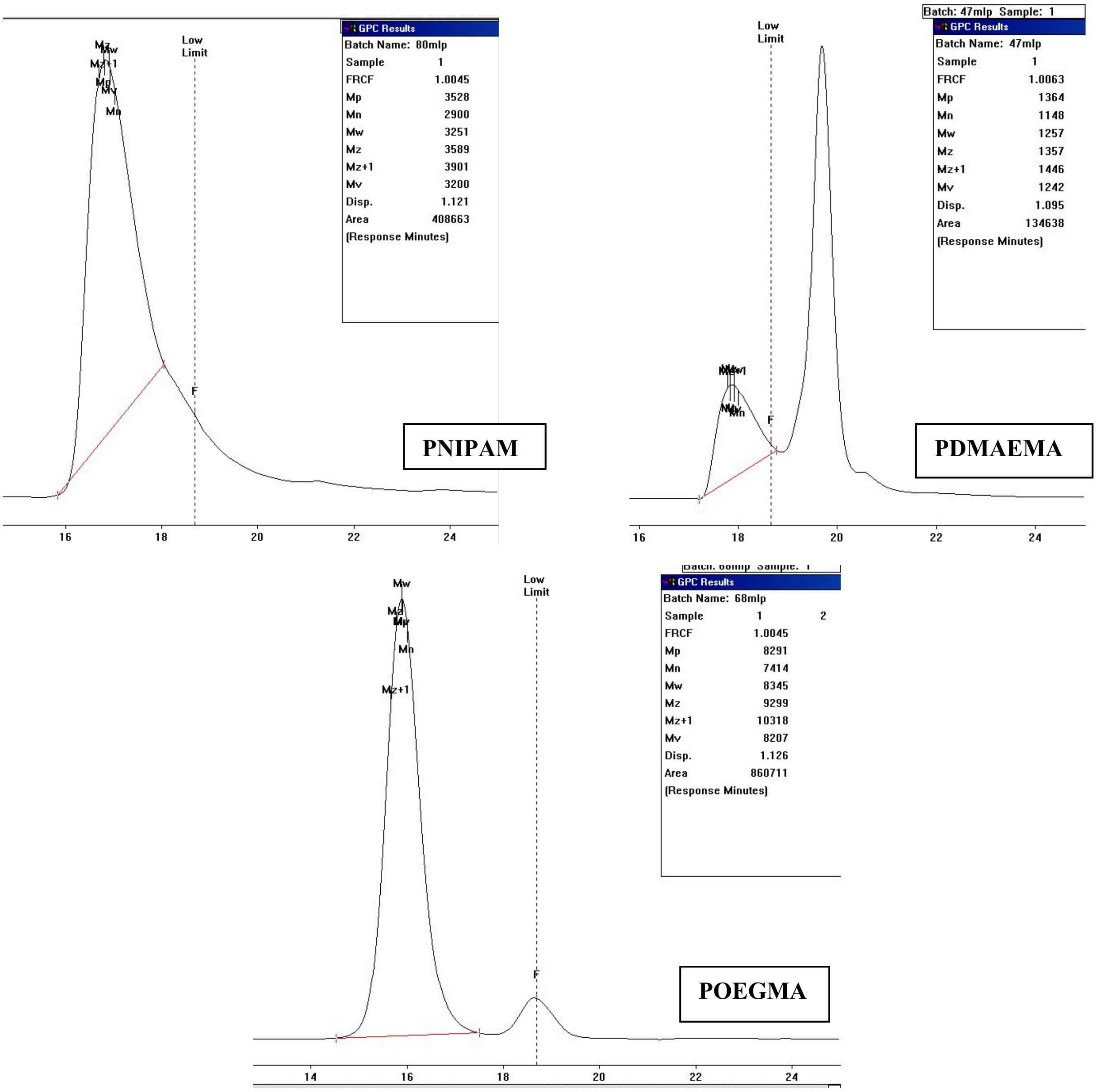

Figure S1. Gel permeation chromatography (GPC) results of synthetic polymers

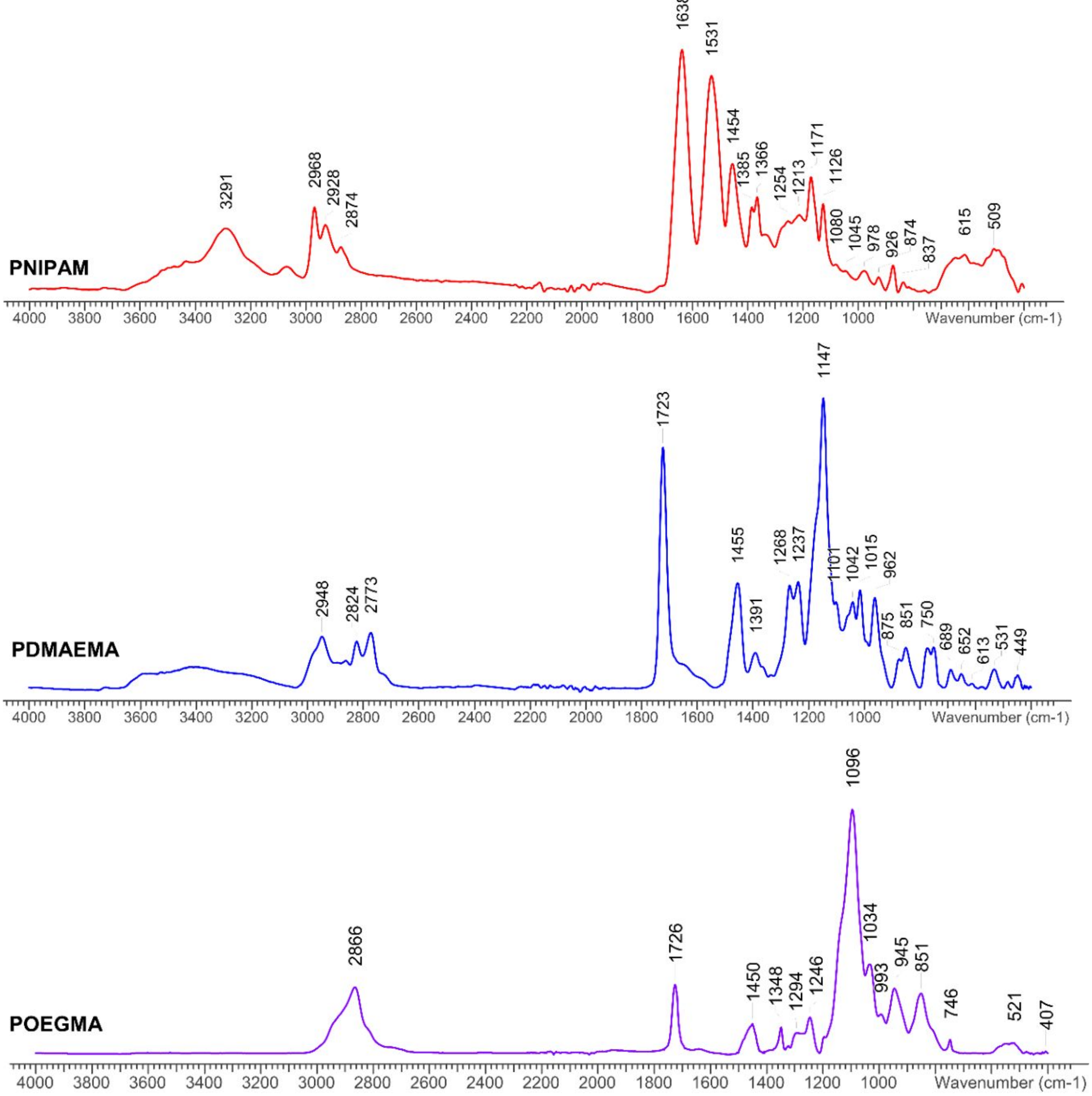

Figure S2. ATR-FTIR spectra of the synthetic polymers

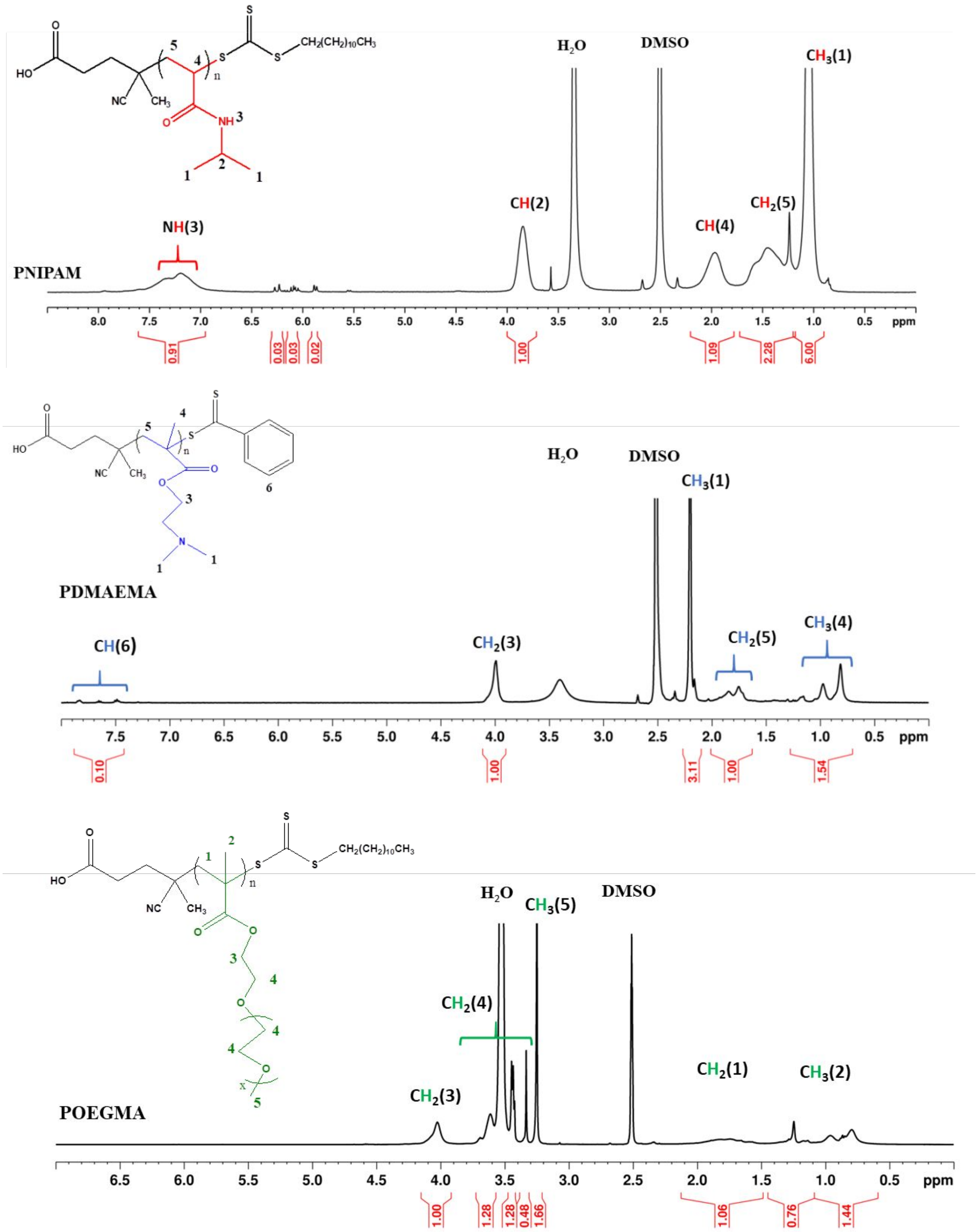

Figure S3. ^{1}H-NMR spectra of synthetic polymers used for the "grafting to" reactions.

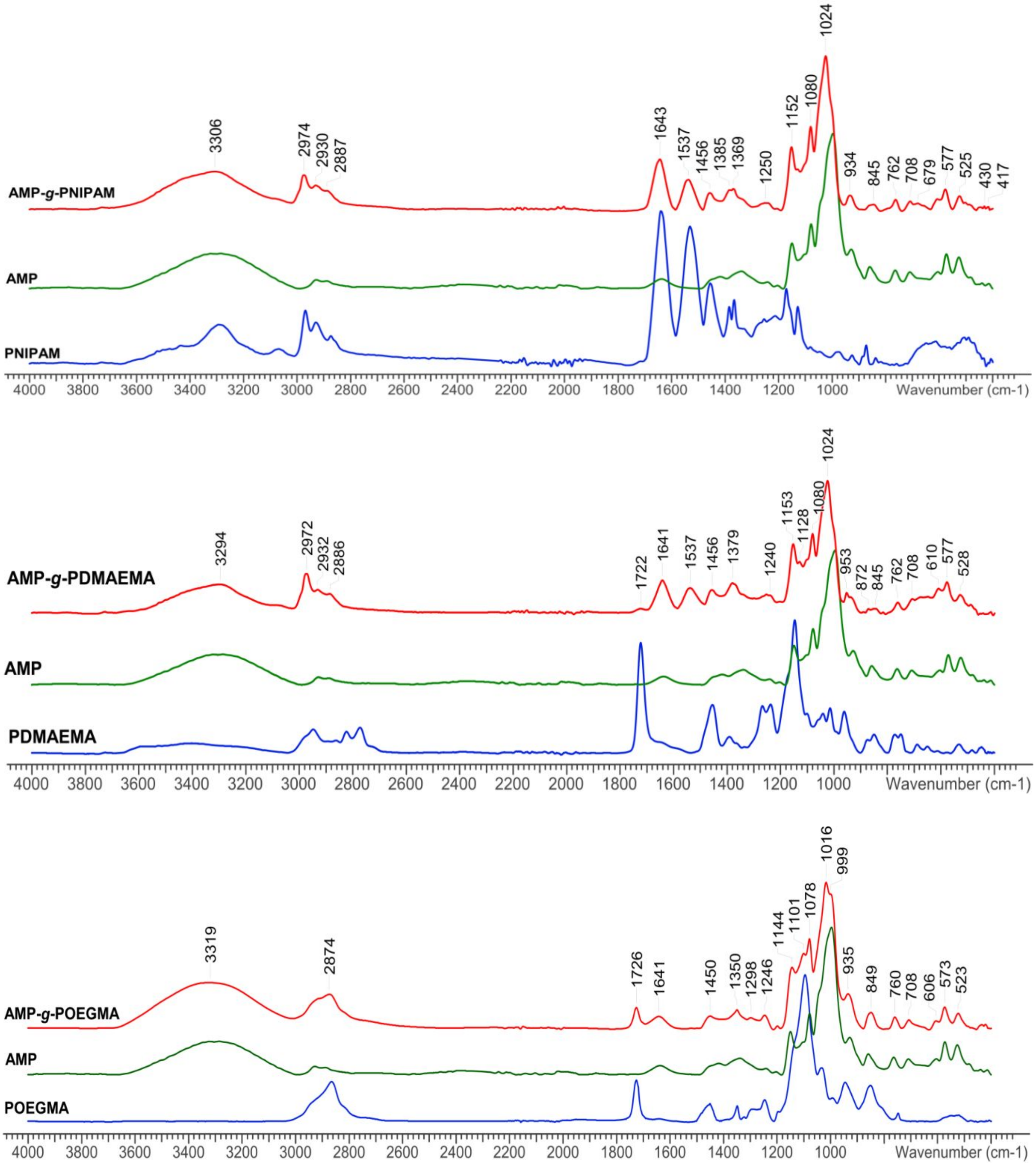

Figure S4. ATR-FTIR spectra of synthetic polymers, AMP and graft copolymers.
